# Comparison of the immunogenicity and safety among COVID-19 vaccines ChadOx-1, CoronaVac and BNT162b2 in systemic lupus erythematosus (SLE) patients: a prospective cohort

**DOI:** 10.1186/s42358-025-00498-9

**Published:** 2025-11-22

**Authors:** Priscila Dias Cardoso Ribeiro, Flavia Maria Matos Melo Campos Peixoto, Edgard Torres dos Reis-Neto, Gecilmara Salviato Pileggi, Nancy Cristina Junqueira Bellei, Marcelo de Medeiros Pinheiro, Vanessa de Oliveira Magalhães, Erika Biegelmeyer, André Gustavo Cunha Trolese, Alexandre Wagner Silva de Souza, Cristiane Kayser, Valéria Valim, Ketty Lysie Libardi Lira Machado, Maressa Barbosa Beloni Lirio, Juliana Ribeiro de Oliveira, Andrea Teixeira de Carvalho, Rodrigo Poubel Vieira de Rezende, Ana Karla Guedes de Melo, Rejane Maria Rodrigues de Abreu Vieira, Vitor Alve Cruz, Viviane Angelina de Souza, Gilda Aparecida Ferreira, Sandra Lúcia Euzébio Ribeiro, Odirlei Monticielo, Ricardo Machado Xavier, Natalia Sarzi Sartori, Emilia Inoue Sato

**Affiliations:** 1 Division of Rheumatology, Department of Medicine, Escola Paulista de Medicina, Universidade Federal de São Paulo (EPM/Unifesp), São Paulo, SP Brazil; 2 Division of Infectology, Department of Medicine, Escola Paulista de Medicina, Universidade Federal de São Paulo (EPM/Unifesp), São Paulo, SP Brazil; 3 Hospital Universitário Cassiano Antônio Moraes (HUCAM), Universidade Federal do Espírito Santo (UFES), Vitória, ES Brazil; 4 Grupo Integrado de Pesquisas em Biomarcadores, Instituto René Rachou, Fundão Oswaldo Cruz (FIOCRUZ-Minas), Belo Horizonte, MG Brazil; 5 Universidade Federal Fluminense, Rheumatology, Niteroi, RJ Brazil; 6 Hospital Universitário Lauro Wanderley, Universidade Federal da Paraíba (UFPB), João Pessoa, PB Brazil; 7 Hospital Geral de Fortaleza (HGF), Universidade de Fortaleza (UNIFOR), Fortaleza, CE Brazil; 8 Faculdade de Medicina, Universidade Federal de Goiás (UFG), Goiânia, GO Brazil; 9 Faculdade de Medicina, Universidade Federal de Juiz de Fora, Juiz de Fora, MG Brazil; 10 Locomotor System Department, Faculdade de Medicina, Universidade Federal de Minas Gerais (UFMG), Belo Horizonte, MG Brazil; 11 Escola de Medicina, Universidade Federal do Amazonas (UFAM), Manaus, AM Brazil; 12 Division of Rheumatology, Department of Internal Medicine, Hospital de Clínicas de Porto Alegre, Universidade Federal Do Rio Grande Do Sul, Porto Alegre, RS Brazil

**Keywords:** Systemic lupus erythematosus, COVID-19 vaccine, Immunogenicity, Vaccine, Adverse event, CoronaVac, ChAdOx1, BNT162b2 Vaccine

## Abstract

**Background:**

The immune response and safety using different COVID-19 vaccine platforms in patients with immune mediated rheumatic diseases is still uncertain. The objective of this study is to compare the immunogenicity and safety after two doses of BNT162b2, CoronaVac and ChadOx-1 in SLE patients.

**Methods:**

Prospective study including SLE patients who received a primary schedule to COVID-19 vaccination between May and August 2021. Immunogenicity, events supposedly attributable to vaccination or immunization (ESAVI) and disease activity were assessed at baseline and after each vaccine dose.

**Results:**

121 SLE patients were included in the cohort, 88 in the immunogenicity analysis and 118 in the safety analysis. The groups were homogenous concerning sex, age, and comorbidities. Seropositivity after two doses of vaccines was similar between CoronaVac (68%), ChadOx1 (80,6%) and BNT162b2 (88%) (p=0.231). However, CoronaVac and ChadOx-1 presented lower titers in comparison with BNT162b2.  Regarding ESAVI, the most frequent reported following first and second vaccine doses were, respectively: injection site pain (65.2%/41.1%), headache (50.9%/29.9%) and arthralgia (37.5%/22.5%). Fever and myalgia were more related to ChAdOx1 than CoronaVac (23.3 vs. 5.0%; p=0.025). There was no difference in MEX-SLEDAI between vaccine platforms. No serious ESAVI were reported.

**Conclusion:**

After two doses, the three COVID-19 vaccine platforms induced a significant increase in antibody titers against SARS-CoV-2. Patients who received BNT162b2 exhibited a higher serological response compared to the other vaccines. All three vaccine platforms demonstrated a favorable safety profile, with no serious ESAVI or worsening of disease activity.

**Clinical trial Number:**

The study was registered in The Brazilian Registry of Clinical Trials (ReBEC) in 04/14/2021 with code RBR-108fyykd.

**Supplementary information:**

The online version contains supplementary material available at 10.1186/s42358-025-00498-9.

## Introduction

Systemic lupus erythematosus (SLE) comprises a complex disease with pleomorphic manifestations with higher morbidity and mortality than the general population that requiring special care regarding infection risk [1–3]. According to a retrospective population-based study carried out in Rio de Janeiro/Brazil, with 2,200 patients with systemic connective tissue diseases who died from 2006 to 2018, infections were the main cause of mortality, corresponding to 53.7% of certificates of death, followed by cardiovascular (37.4%) and respiratory (30.3%) diseases [3]. The susceptibility to infection can result from immunosuppressants and glucocorticoids (GCs) [4–7], especially in doses equivalent to prednisone ≥ 10 mg/day [8]. In addition, disease activity, which is associated with acquired and transient immune system dysfunction, and typical immunophenotypic alterations, such as an increased number of peripheral Th17 cells and an increased number of low-density granulocytes [9], as well as deficiencies and/or consumption of complement fractions, such as C1q and C4, highly associated with SLE [10], also contribute to an increased risk of infection [6, 9, 11–13]. Finally, the comorbidity profile of SLE patients, especially in the presence of chronic kidney failure or lung disease, also increases the infectious risk [14, 15].

Since the onset of the SARS-CoV-2 pandemic in 2020, the heightened vulnerability of patients with systemic lupus erythematosus (SLE) to infections has become an issue of particular concern. Emerging evidence has demonstrated that this population is at increased risk of hospitalization associated with COVID-19. Ethnicity, comorbidities (such as cardiovascular disease and kidney failure), and higher body mass index were identified as independent predictors of hospitalization [16]. In addition, immunosuppressants commonly used to treat SLE were associated with an increased risk of death from COVID-19 [17].

Vaccination against COVID-19 has been the most impactful strategy in reducing the morbidity and mortality caused by the virus. To date, over 13.5 billion doses have been administered worldwide [18]. In phase III trials, vaccination with at least two doses of the BNT162b2 (Pfizer/BioNTech), mRNA01273 (Moderna), ChAdOx1 nCoV-19/AZD1222, CoronaVac (Sinovac/Butantan) and single-dose Ad26.COV2.S vaccines (Janssen/Johnson & Johnson) have been proven to be safe and to reduce the risk of severe SARS-CoV-2 infection and its complications in the general population [19–23].

However, it is well established that multiple factors influence the magnitude and duration of the immune response to vaccination. These include vaccine-related factors, such as the type of antigen, route of administration, dosage, vaccination schedule, and the use of adjuvants. Additionally, individual-specific factors, such as age (e.g., immunosenescence), underlying health conditions, comorbidities, and immunosuppressive therapies further influence this response [24, 25]. Thus, despite SLE affects predominantly young individuals, the disease’s inherent immunodeficiencies along with the immunosuppressive drugs used to manage its activity, can compromise the response to these vaccines.

Data suggest that rituximab [26–30] glucocorticoids [26, 27, 29–32], methotrexate [26, 27, 33] and mycophenolate mofetil [26, 29–32] are the drugs most associated with reduced immunogenicity of vaccines (mRNA platforms and inactivated virus) for SARS-CoV-2. However, the GC dose associated with reduced vaccine response is still controversial. Furthermore, it is not known whether there is a difference in the impact of immunosuppressants and immunomodulators on vaccine response in different platforms.

The aim of this study is to evaluate serological response and events supposedly attributable to vaccination or immunization (ESAVI) between CoronaVac (Sinovac/Instituto Butantan), ChadOx-1 (Fiocruz/AstraZeneca) and BNT162b2 (Pfizer/BioNTech) vaccines in a cohort of SLE patients.

## Methods

### Study design

**Fig. 1:**
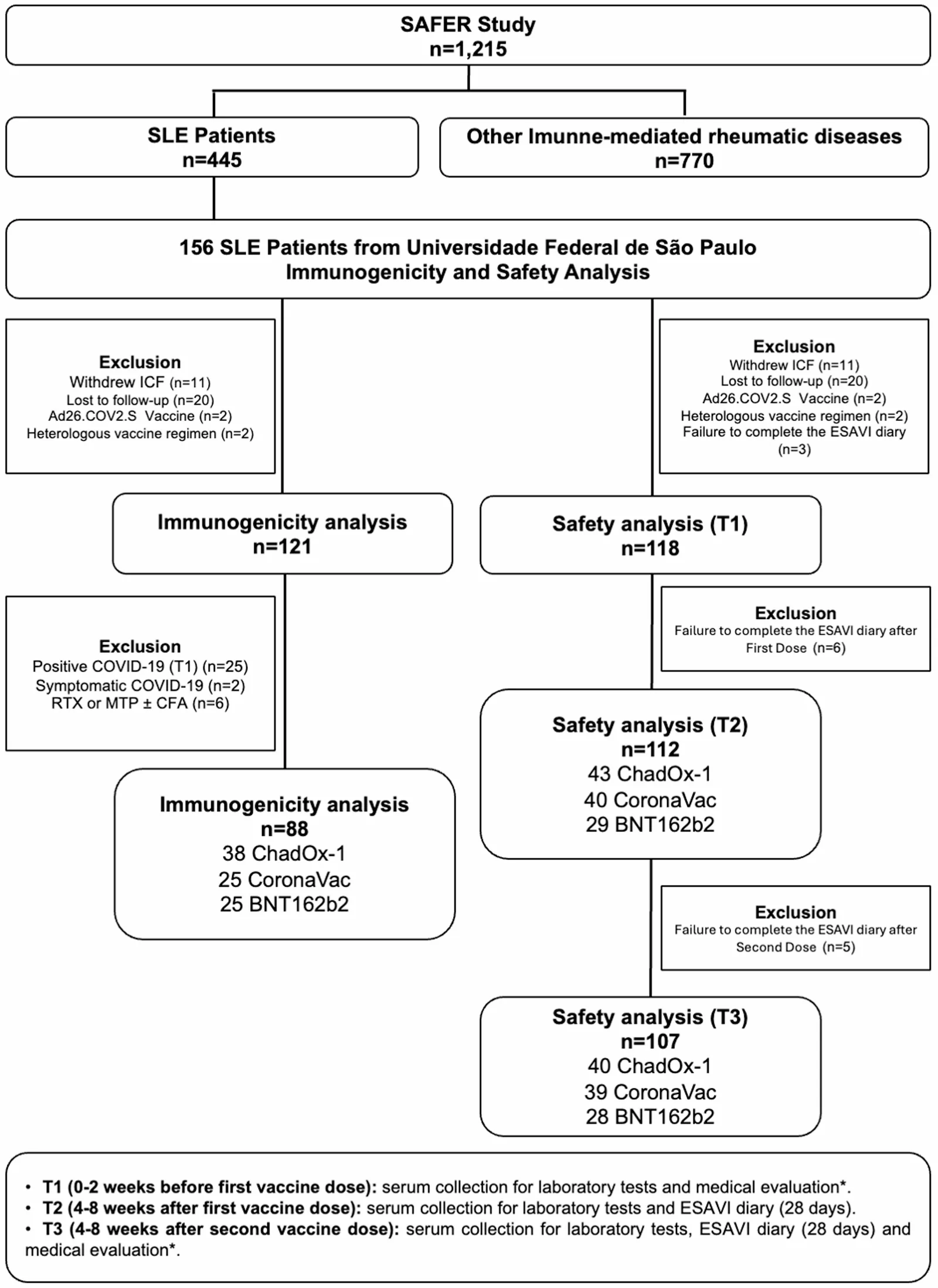
Recruitment, follow-up and evaluations of SLE patients in the study according to vaccines. *Medical evaluation included assessment of disease activity and occurrence of events supposedly attributable to vaccination or immunization. SLE: Systemic lupus erythematosus. ICF: Informed consent form. RTX: Rituximab. MTP: methylprednisolone pulse therapy. CFA: Cyclophosphamide. ESAVI: events supposedly attributable to vaccination or immunization

SAFER Study (*Safety and Effectiveness on COVID-19 Vaccine in Rheumatic Disease)* is a prospective multicenter, observational, longitudinal, phase 4, real-life study carried out in Brazil which assessed safety and effectiveness of SARS-Cov-2 vaccines in patients with immune-mediated rheumatic diseases (IMRDs). The present study was focused in evaluating safety, the effect on disease activity and the immunogenicity of the vaccines BNT162B2 (BNT162b2), CoronaVac (SinoVac) and ChAdOx1 (ChAdOx1) in a single-center prospective cohort of SLE patients (Fig. 1). The study was registered in The Brazilian Registry of Clinical Trials (ReBEC) in 04/14/2021 with code RBR-108fyykd.

### Patients

SLE patients from the rheumatology unit of a tertiary university hospital in São Paulo, Brazil, aged over 18 years, who met the 2019 ACR/EULAR criteria (43), received two doses of the same vaccine platform between May and August 2021, and signed the Informed Consent Form (ICF) approved by the Institutional Ethics Committee were included as a convenience sample. The study was conducted in accordance with the Declaration of Helsinki and was reviewed and approved by the coordinating center’s ethics committee (Research Ethics Committee of the Universidade Federal de São Paulo, CAAE: 43479221.0.1001.5505).

Exclusion Criteria: other causes of immunosuppression (people living with the Human Immunodeficiency Virus; CD4 < 200 cells/mm^3^; organ transplant recipients; primary immunodeficiency; neoplasia in the last 5 years; previous history of thymus diseases, such as myasthenia gravis, thymoma, thymus aplasia or its surgical removal; pregnancy; and a history of serious adverse reaction to any previously administered vaccine. For the immunogenicity analysis, were also excluded: patients who had received a heterologous SARS-CoV-2; a single dose of Ad26.COV2.S (Janssen/Johnson & Johnson); blood transfusion in the last 30 days; plasmapheresis or human immunoglobulin in the 30 days; rituximab in the last 20 weeks; pulse therapy with cyclophosphamide in the last 3 weeks; or, another inactivated vaccine in the last 14 days or a live virus vaccine in the last 28 days or have had suspicion or laboratory diagnosis (RT-PCR or serology or rapid test) of SARS-CoV-2 infection in the last 4 weeks.

Patients were classified according to immunosuppression degree according to the criteria proposed by the Brazilian Societies of Rheumatology, Dermatology and Study Groups on Inflammatory Bowel Diseases [34]: 1) Non-immunosuppressed: not using any medication or using only sulfasalazine, hydroxychloroquine, topical or inhaled corticosteroids, periarticular or intra-articular glucocorticoids. 2) Low degree of immunosuppression: use of methotrexate ≤0.4 mg/kg/week or at a dose ≤20 mg/week; leflunomide 20 mg/day; prednisone or equivalent ≤ 20 mg/day. 3) High degree of immunosuppression: GC ≥ 20 mg/day (prednisone or equivalent), for a period ≥ 14 days; pulse therapy with methylprednisolone; mycophenolic acid; cyclosporine; cyclophosphamide; tacrolimus; azathioprine; janus kinase inhibitor (JAKI); and other b-DMARDs (biological disease-modifying antirheumatic drugs). In the immunogenicity analysis prednisone dose was categorized in ≤ 5 mg/day, 6–10 mg/day and ≥ 11 mg/day and in the safety analysis < 7.5 mg/day and ≥ 7.5 mg/day.

### Vaccination

Patients received the vaccines made available by the Brazilian Ministry of Health during the period of the study, according to the national program of immunization (NPI): BNT162b2, CoronaVac, ChAdOx1 or Ad26.COV2-S. For this study, those who received BNT162B2, CoronaVac and ChAdOx1 were non-randomly selected.

### Follow-up

Before receiving the first dose of one of the vaccines (T1), patients were assessed for disease activity through MEX-SLEDAI [35] and a blood sample was collected for immunogenicity evaluation. During this first appointment, a vaccination diary was also provided for the patient to complete within 28 days, regarding any complaint of an ESAVI. Four to eight weeks after the first dose of one of the vaccines (T2), all patients were re-assessed for ESAVI following the first vaccination and a new blood sample was collected. A second dose of the vaccine was applied, and a new vaccination diary was made available to the patient to be completed regarding any possible ESAVI. After 4–8 weeks after the second dose (T3) of either vaccine, patients were re-assessed for new post-vaccination ESAVI as well as disease activity, medications and new blood samples (Fig. 1).

### Laboratory analysis

At each visit (T1, T2 and T3) serological vaccine response was assessed by a chemiluminescent immunoassay (Anti-RBD IgG, ACCESS SARS-CoV-2 IgG/IgG, Beckman Coulter, California, USA). The kit was already approved in Brazil (ANVISA registration N 10033121020) and in European Union (CE Mark) with sensitivity of 100% (92.7–100%) and specificity of 99.8% (99.4–99.9%), without cross-reactivity (Kit package insert, May 2021).

### Data analysis and statistics

All data were maintained on the Redcap Platform, hosted on the server of the Sociedade Brasileira de Reumatologia (SBR) and were analyzed using IBM-SPSS v.25.0 (Statistical Package for Social Sciences) software. Data was described as mean, median, standard deviation, minimum and maximum values for quantitative variables and as frequencies (absolute and relative) for qualitative variables. Normality of quantitative variables was assessed by Kolmogorov-Smirnoff test. Parametric and nonparametric tests, univariate and multivariate analysis were used for statistical analysis. Student’s t-test or Mann-Whitney test were used to compare continuous variables according to distribution. Association of participant characteristics with received vaccines and responses to vaccination was verified using Chi-square, exact Fisher’s or Kruskal-Wallis tests. Multiple comparisons (post-hoc) were performed using Bonferroni method to correct the significance level. The correlation between response to vaccination and MEX-SLEDAI [35] was verified using Spearman’s correlation coefficient. Multiple linear regression model was adjusted to estimate the expected mean response value according to selected characteristics. The significance level adopted was 5%.

## Results

One hundred and fifty-six eligible SLE patients were recruited. Thirty-five were excluded from all the analysis due to losing follow-up, other vaccine regimens or withdrawing their ICF. Besides that, regarding immunogenicity analyses, 27 patients were excluded due to seropositivity prior to vaccination (T1) or proven COVID-19 infection between T1 and T3. Patients using rituximab (*n* = 1), methylprednisolone pulse therapy (*n* = 4) and/or cyclophosphamide (*n* = 5) were also excluded (all had received CoronaVac). Concerning safety analysis, a total of 118 SLE patients were included. However, due to incomplete ESAVI diaries following the first and second doses of the vaccines, a few patients were excluded for T2 and T3 evaluations (Fig. 1).

There were 16 confirmed cases of SARS-CoV-2 infection occurring more than 4 weeks after the first vaccine dose, of which twelve were seroreactive at baseline. Among the 73 patients who reported no prior infection, 13 were seroreactive at T1. None of the patients who were uncertain about prior infection were seroreactive at T1. Analysis of whether prior SARS-CoV-2 exposure before the vaccination schedule was associated with greater post-vaccination immunogenicity revealed no difference.

### Clinical and demographic data

Regarding the 88 patients who participated in the immunogenicity study, 90,9% were female; the mean age was 36.5 ± 10.9 years with mean disease duration was 11.0 ± 8.4 years. Thirty-eight (43.2%) participants had received ChadOx-1 vaccine, 25 (28.4%) received CoronaVac and 25 (28.4%) received BNT162b2. The groups were homogenous and comparable in most variables, except for the presence of comorbidities, with pulmonary disease was more frequent in the BNT162b2 group (*p* = 0.011), and disease duration longer among patients in the ChadOx-1 group (13.5±6.7) compared to the CoronaVac (*p* = 0.003) and BNT162b2 (*p* = 0.018) groups. There was no difference regarding GC dose, immunosuppressants and antimalarials between the three groups (Table 1).

**Table 1:** Clinical and demographic data SLE patients included in the immunogenicity and safety analysis n (%)

Characteristics	Immunogenicity analysis					Safety analysis				p
	Total 88	Vaccine			p	Total 118	Vaccine			
		ChAdOx1	CoronaVac	BNT162b2			ChAdOx1	CoronaVac	BNT162b2	
		38 (43.2%)	25 (28.4%)	25 (28.4%)			45 (38%)	44 (37%)	29 (24%)	
Age^ϒ^	36.5±10.9	38.3±9.8	33.3±12.6	36.9±10.6	0.104 ^c^	36.6±11.3	38.2±9.5	34.3±13.3	37.5±10.1	0.074^c^
Female	80 (90.9)	32 (84.2)	24 (90.3)	24 (96)	0.242 ^c^	104 (88.1)	39 (86.7)	37 (84.1)	28 (96.6)	0.261^a^
Race										
White	33 (37.5)	11 (38.5)	15 (60)	7 (28)	0.170 ^c^	41 (35.3)	12 (27.3)	20 (46.5)	9 (31.0)	0.271^b^
Black	13 (14.8)	7 (18.4)	3 (12)	3 (12)		20 (17.2)	9 (20.5)	8 (18.6)	3 (10.3)	
Brown	39 (44.3)	18 (47.4)	7 (28)	14 (56)		52 (44.8)	21 (47.7)	15 (34.9)	16 (55.2)	
Yellow	3 (3.4)	2 (5.3)	0 (0)	1 (4)		3 (2.6)	2 (4.5)	0 (0)	1 (3.4)	
Anti-dsDNA	41 (46.6)	16 (48.5)	13 (68.4)	12 (48)	0.356 ^b^	58 (52.7)	19 (15.2)	23 (59)	58 (52.7)	0.441^a^
MEX-SLEDAI^ϒ^	2.9±3.3	2.3±2.9	3.5±4.1	3.0±2.9	0.516 ^a^	3.2±3.7	2.5±2.9	3.9±4.6	3.3±3.1	0.444^a^
Anti-malarial^δ^	70 (79.5)	28 (73.7)	20 (80)	22 (88)	0.42 ^b^	94 (79.7)	34 (75.6)	35 (79.5)	25 (86.2)	0.546^b^
Oral Glucocorticoid	33 (37.5)	14 (36.8)	9 (36)	10 (40)	> 0.99 ^b^	47 (39.8)	17 (37.8)	17 (38.6)	13 (44.8)	0.838^b^
Prednisone dose (mg/day)	10.0±7.0	6.6±3.2	15±10.1	10.2±5.8	0.101^a^	13,2 ±10	7,5±4,3	21,7±15,9	9,8±5,2	0,006^a^
Methylprednisolone pulse therapy	-	-	-	-	-	2 (1.7)	1 (2.2)	1 (2.3)	0 (0.0)	> 0.99^c^
Immunosuppressive drugs										
Azathioprine	27 (30.7)	11 (28.9)	8 (32)	8 (32)	> 0.99 ^b^	35 (29.7)	12 (26.7)	13 (29.5)	10 (34.5)	0.790^b^
Tacrolimus	2 (0.2)	1 (2.6)	0 (0)	1 (4)	> 0.99 ^c^	1 (0.8)	0 (0.0)	0 (0.0)	1 (3.4)	0.246^c^
Leflunomide	3 (0.3)	2 (5.3)	0 (0)	1 (4)	0.789 ^c^	5 (4.2)	3 (6.7)	1 (2.3)	1 (3.4)	0.645^c^
Methotrexate	13 (14.8)	7 (18.4)	1 (4)	5 (20)	0.204 ^c^	15 (12.7)	7 (15.6)	2 (4.5)	6 (20.7)	0.103^b^
Mycophenolate	26 (29.5)	12 (31.6)	7 (28)	7 (28)	0.914 ^b^	28 (23.7)	13 (28.9)	8 (18.2)	7 (24.1)	0.496^b^
Cyclophosphamide pulse therapy	-	-	-	-	-	5 (4.2)	0 (0.0)	5 (11.4)	0 (0.0)	0.019 ^c^
Rituximab	-	-	-	-	-	2 (1.7)	0 (0.0)	2 (4.5)	0 (0.0)	0.196^c^
Level of Immunosuppression										
No	16 (18.2)	6 (15.8)	7 (28)	3 (12)		20 (16.9)	8 (17.8)	9 (20.5)	3 (10.3)	
Low	15 (17)	8 (21.1)	2 (8)	5 (20)	0.458 ^b^	2 (1.7)	2 (4.4)	0 (0.0)	0 (0.0)	0.448^b^
High	57 (64.8)	24 (63.2)	16 (64)	17 (68)		96 (81.4)	35 (77.8)	35 (79.5)	26 (89.7)	
COVID Infection Pre-Vaccine	-	-	-	-	-	23 (20.0)	4 (9.3)	11 (25.6)	8 (27.6)	0.084^a^

^γ^ Mean±Standard Deviation. ^δ^Hydroxychloroquine or Chloroquine Diphosphate. ^a^Kruskal-Wallis test; ^b^Chi-square test; ^c^Fisher’s Exact Test

Concerning safety analysis, 118 SLE patients were included. Most were females [104 (88.1%)], with a mean age of 36.6 ± 11.2 years and disease duration of 11.1 ± 8.7 years. Seventy-five patients (63.5%) declared themselves non-white. Twenty-three (19.5%) declared previous infection with the SARS-CoV-2 virus before the first dose of the vaccine, of which 15 (65.2%) presented a positive result in the serological test. Almost 80% were using hydroxychloroquine, 47 (39.8%) glucocorticoids and 95 (80.5%) were using some other immunosuppressant, with azathioprine, mycophenolate and methotrexate being the most used. Of the 47 patients who were taking glucocorticoids, 29 (61.7%) were on daily prednisone ≥ 7.5 mg/day. Ninety-six (81.4%) patients were under high degree of immunosuppression (Table 1).

### Immunogenicity analysis

Seropositivity after the second dose, stratified by vaccine platform, was 80% for ChAdOx-1, 68% for CoronaVac, and 88% for BNT162b2 (*p* = 0.23). Univariate analysis, including the assessment of age, sex, and comorbidities, revealed no significant factors associated with the frequency of vaccine response (Table 2). However, a trend toward a lower MEX-SLEDAI index was observed among responding patients (*p* = 0.066), regardless of the vaccine platform used (Table 2). A decreasing numerical response rate (not statistically significant) was observed according to the degree of immunosuppression, being higher among non-immunosuppressed individuals and lower among those with a high degree of immunosuppression (Table 2).

**Table 2:** Seropositivity rate after COVID-19 vaccines according to demographic and clinical factors n (%)

Characteristics		Seropositivity rate		p
		Non-Responders N = 20	Responders N = 68	
Disease duration^δ^		12.8±8.9	10.4±8.1	0.241****
MEX-SLEDAI^δ^		4.5±4.5	2.5±2.9	0.066****
Vaccine	ChadOx-1	7 (19.5)	29 (80.5)	0.231*
	CoronaVac	8 (44.4)	17 (68.0)	
	BNT162b2	3 (16.7)	22 (88.0)	
Heart disease		2 (33.3)	4 (66.7)	0.601**
Lung disease		0 (0.0)	4 (100)	0.575**
CKD (GFR < 60 ml/min)		0 (0.0)	1 (100)	> 0.99**
Hypertension		4 (19.1)	17 (80.9)	> 0.99**
Obesity		1 (20.0)	4 (80.0)	> 0.99**
Hydroxychloroquine		12 (17.6)	56 (82.4)	0.192**
Corticosteroid use n (%)		9 (31.4)	24 (68.6)	0.285*
Prednisone dose	≤ 5 mg/day	2 (14.3)	12 (85.7)	0.409
	6-10 mg/day	4 (36.4)	7 (63.6)	
	≥ 11 mg/day	3 (37.5)	5 (62.5)	
Degree of immunosuppression	No	2 (12.5)	14 (87.5)	0.295**
	Low	3 (20.0)	12 (80.0)	
	High	15 (26.3)	42 (73.7)	

CKD: Chronic kidney disease. GFR: glomerular filtration rate. δMedia ± Standard deviation. *Chi-square test. ** Fisher’s Exact Test. *** T test. **** Man-Whitney test

**Fig. 2:**
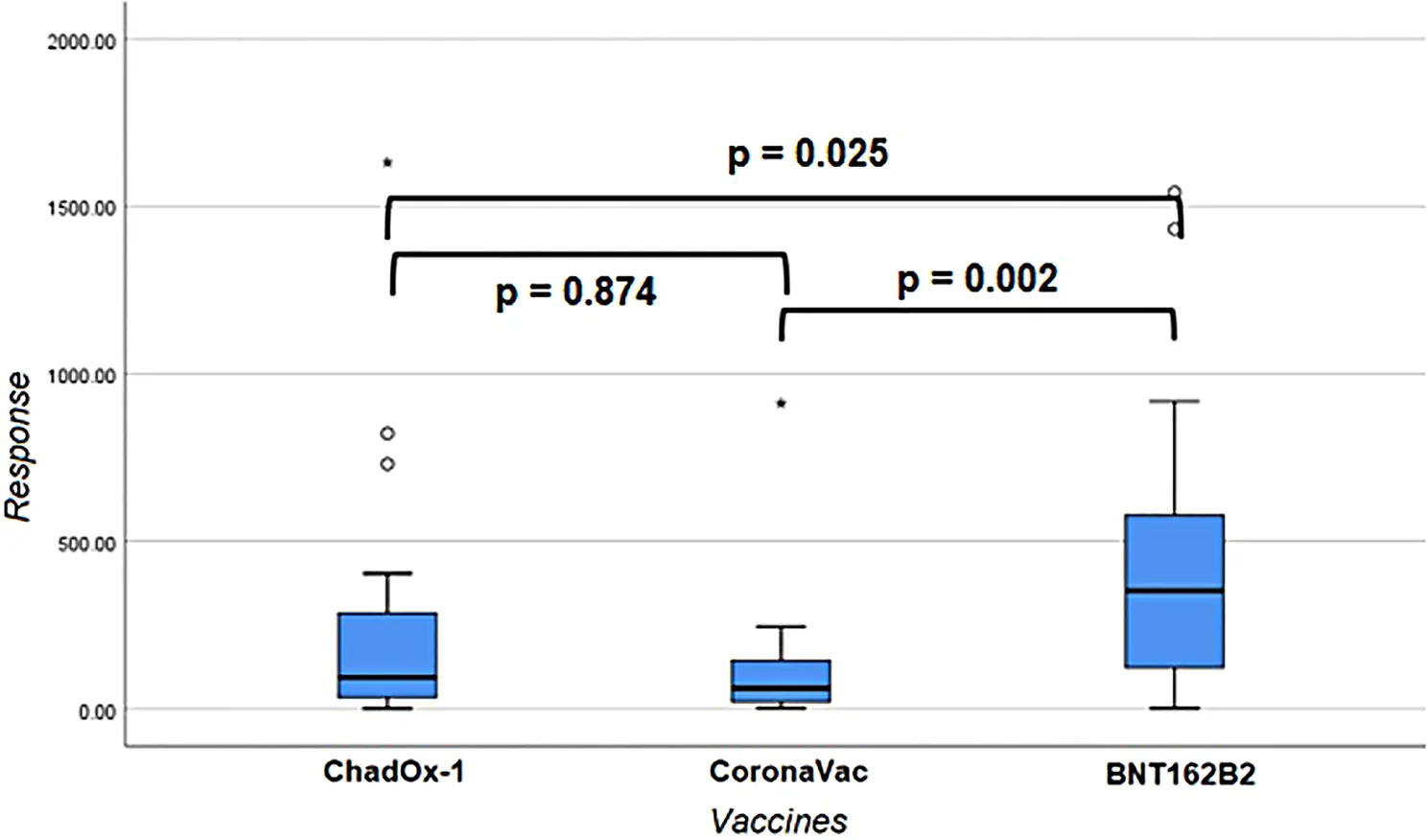
Anti-RBD titers among patients vaccinated with ChadOx-1, CoronaVac and BTN162b2

When vaccine response was evaluated by anti-RBD titers, those were significantly higher in the BNT162b2 group compared to CoronaVac (436.1±416.0 vs 114.1 ± 183.2; *p* = 0.002) and to ChadOx-1 (436.1±416.0 vs 199.3 ± 311.7; *p* = 0.025) (Table 3 and Fig. 2). No association was observed between anti-RBD antibody titers and variables including sex, comorbidities, use of hydroxychloroquine, glucocorticoid dosage, or degree of immunosuppression.

**Table 3:** Anti-RBD titers after COVID-19 vaccine according demographic and clinical factors

Characteristics		After 2nd dose	
		Anti-RBD IgG (titers)	
		ρ (p)	
Age ρ (p)^δ^		0,014 (0,897)	
Disease duration ρ (p)^δ^		−0,139 (0,203)	
MEX-SLEDAI ρ (p)^δ^		−0,036 (0,746)	
		med (mín; max)	p
Vaccine	ChadOx1	93.8 (0.7; 1630.9)	0.002**
	CoronaVac	61.2 (1.7; 911.9)	
	BNT162B2	352.7 (1.6; 1541.2)	
Prednisone dose	≤ 5 mg/day	111.0 (6.2; 1630.9)	0.115**
	6–10 mg/day	64.2 (1.03; 917.4)	
	≥ 11 mg/day	39.9 (1.6; 196.9)	
Degree of immunosuppression	No	101.5 (11.3; 419.3)	0.945**
	Low	73.4 (2.5; 1431.9)	
	High	73.7 (0.67; 1630.9)	

^δ^Media ± Standard deviation.**Kruskal-Wallis Test. ρ: Spearman’s correlation coefficient

In the multivariate analysis evaluating the combined effect of the vaccine and GC use on anti-RBD titers, the linear regression model showed a lower mean titer for those vaccinated with ChadOx-1 (β = −245.7; *p* = 0.02) and CoronaVac (β = −293.8; *p* = 0.01) compared to BNT162b2 (used as a reference) (Table 4). A trend towards an increase in the serological response among non-users of GC and those using up to prednisone 5 mg/day was observed compared to those with prednisone ≥ 11 mg/day (β = 216.35; *p* = 0.06) (Table 4).

**Table 4:** Generalized linear models to evaluate induced antibody titers (Anti-RBD) after the COVID-19 vaccine in all patients (Model 1) and only in corticosteroid users (Model 2)

Predictor variables		Model 1	p
		β [IC 95%] (min; max)	
Vaccines	ChAdOx-1	−245.7 (−400.8; −90.6)	0.02
	CoronaVac	−293.8 (−466.5; −121.0)	0.01
	BNT162b2	Reference	
Prednisone dose	Non users or users of ≤ 5 mg/day	216.35 (−14.3; 447.1)	0.06
	6 - 10 mg/day	122.9 (−162.7; 408.5)	0.39
	≥11 mg/day	Reference	
Degree of immunosuppression	No	−139,25 (−311.65; 33.1)	0.11
	Low	−73,46 (−252.65; 105.7)	0.42
	High	Reference	

Predictor variables		Model 2 - GC Users	p
		β [IC 95%] (min; max)	
Vaccine	ChadOx-1	Reference	
	CoronaVac	−23.3 (−435.9; 389.4)	0.909
	BNT162B2	412.5 (44.9; 780.1)	0.029
GC dose	≤5 mg/day	Reference	
	≥6 a 10 mg/day	−331.9 (−686.5; 22.5)	0.065
	≥11 mg/day	−427.1 (−854.7; 0.58)	0.05

GC: glucocorticoid

### Events supposedly attributable to vaccination or immunization (ESAVI)

Events supposedly attributable to vaccination or immunization, local or systemic, were more frequent after the first dose of any of the vaccines and both were less common with CoronaVac (47.5%, *p* = 0.001; and 57.5%, *p* = 0.029; respectively). After the second dose, only local ESAVI with CoronaVac (28.2%, *p* = 0.047) were significant. Those who received ChAdOx1 experienced more systemic symptoms after the first dose (83.7%), which was not observed after the second dose (Table 5).

**Table 5:** Events supposedly attributable to vaccination or immunization following the first and second COVID-19 vaccine dose in SLE patients, according to different platforms N (%)

ESAVI	After First dose					After Second dose				
	Total	ChAdOx1	CoronaVac	BNT162b2	p	Total	ChAdOx1	CoronaVac	BNT162b2	p
	112	43 (42.8)	40 (35.7)	29 (25.8)		107	40 (37.3)	39 (36.4)	28 (26.1)	
Local Reactions Erythema	14 (12.5)	8 (18.6)	4 (10.0)	2 (6.9)	0.298^a^	8 (7.5)	4 (10.0)	2 (5.1)	2 (7.1)	0.895^b^
Bruising	9 (8.0)	4 (9.3)	2 (5.0)	3 (10.3)	0.745^b^	7 (6.5)	2 (5.0)	4 (10.3)	1 (3.6)	0.548^b^
Swelling	29 (25.9)	13 (30.2)	6 (15.0)	10 (34.5)	0.137^a^	12 (11.2)	3 (7.5)	5 (12.8)	4 (14.3)	0.641^b^
Induration	31 (27.7)	12 (27.9)	10 (25.0)	9 (31.0)	0.833^a^	10 (9.3)	4 (10.0)	2 (5.1)	4 (14.3)	0.415^b^
Pain	73 (65.2)	32 (74.4)	18 (45.0)	23 (79.3)	0.003^a^	44 (41.1)	18 (45.0)	10 (25.6)	16 (57.1)	0.03^a^
Skin Rash	17 (15.2)	7 (16.3)	3 (7.5)	7 (24.1)	0.164^a^	5 (4.7)	2 (5.0)	1 (2.6)	2 (7.1)	0.848^b^
Pruritus	7 (41.2)	2 (28.6)	1 (33.3)	4 (57.1)	0.811^b^	2 (40.0)	1 (50.0)	0 (0.0)	1 (50.0)	> 0.99^b^
Systemic reactions Nausea or vomiting	25 (22.3)	12 (27.9)	10 (25.0)	3 (10.3)	0.205^a^	13 (12.1)	8 (20.0)	3 (7.7)	2 (7.1)	0.192^b^
Fatigue	42 (37.5)	20 (46.5)	12 (30.0)	10 (34.5)	0.298^a^	24 (22.4)	9 (22.5)	5 (12.8)	10 (35.7)	0.094^a^
Headache	57 (50.9)	27 (62.8)	17 (42.5)	13 (44.8)	0.139^a^	32 (29.9)	14 (35.0)	9 (23.1)	9 (32.1)	0.533^a^
Myalgia	40 (35.7)	20 (46.5)	8 (20.0)	12 (41.4)	0.031^a^	24 (22.4)	10 (25.0)	6 (15.4)	8 (28.6)	0.412^a^
Arthralgia	42 (37.5)	21 (48.8)	11 (27.5)	10 (34.5)	0.134^a^	27 (25.2)	13 (32.5)	6 (15.4)	8 (28.6)	0.209^a^
Fever	14 (12.5)	10 (23.3)	2 (5.0)	2 (6.9)	0.025^a^	8 (7.5)	4 (10.0)	3 (7.7)	1 (3.6)	0.669^b^
Vertigo	28 (25.0)	11 (25.6)	10 (25.0)	7 (24.1)	> 0.99^a^	17 (15.9)	9 (22.5)	3 (7.7)	5 (17.9)	0.193^a^
Other reactions	39 (34.8)	19 (44.2)	10 (25.0)	10 (34.5)	0.187^a^	20 (18.7)	9 (22.5)	4 (10.3)	7 (25.0)	0.249^a^

^a^Chi-square; ^b^Fisher Test. ESAVI: events supposedly attributable to vaccination or immunization

The most frequent ESAVI after the first dose of the vaccine were local pain (65.2%), headache (50.9%), arthralgia (37.5%) and fatigue (37.5%). SLE patients who received CoronaVac presented less pain at the vaccine site (45.0% vs. 65.2 vs. 74.4%; *p* = 0.031) when compared to other vaccines, and less myalgia than ChAdOx1 (20.0% vs. 46.5, *p* = 0.003). On the other hand, fever was more frequent among those who received ChAdOx1 (23.3% vs. 5.0%; *p* = 0,025) (Table 5).

After the second dose of the vaccines, local pain was reported by 63 (75.2%) participants, followed by headache in 57 (50.9%) and arthralgia in 27 (25.2%). Only pain at the injection was less frequent among those who received CoronaVac when compared to BNT162b2 (25.6% vs. 57.1%; *p* = 0.03) (Table 5).

No significant difference was observed in the duration of events supposedly attributable to vaccination or immunization following the first dose of any of the vaccine platforms. The average duration of events supposedly attributable to vaccination or immunization was longer among participants who reported arthralgia (16.2±11.2 days), myalgia (13.0±11.2 days) and skin rash (11.5± 10.7 days), however, the difference was not significant. (Supplemental Table 1). Regarding events supposedly attributable to vaccination or immunization and Prior COVID infection (pre-exposed), There was no difference in the comparison between local or systemic ESAVI for those SLE patients previously infected with SARS-CoV-2 (pre-exposed) following the first and second doses. (Supplemental Table 2).

The use of hydroxychloroquine did not demonstrate to be a protective factor for the occurrence of ESAVI. Also, there was no difference on ESAVI and the degree of immunosuppression (Supplemental Table 3). The use of prednisone in dose ≥ 7.5 mg/day before the first or second vaccine dose did not make a difference in relation to events supposedly attributable to vaccination or immunization in general, neither when evaluated by vaccine platform (Supplemental Table 4).

**Fig. 3:**
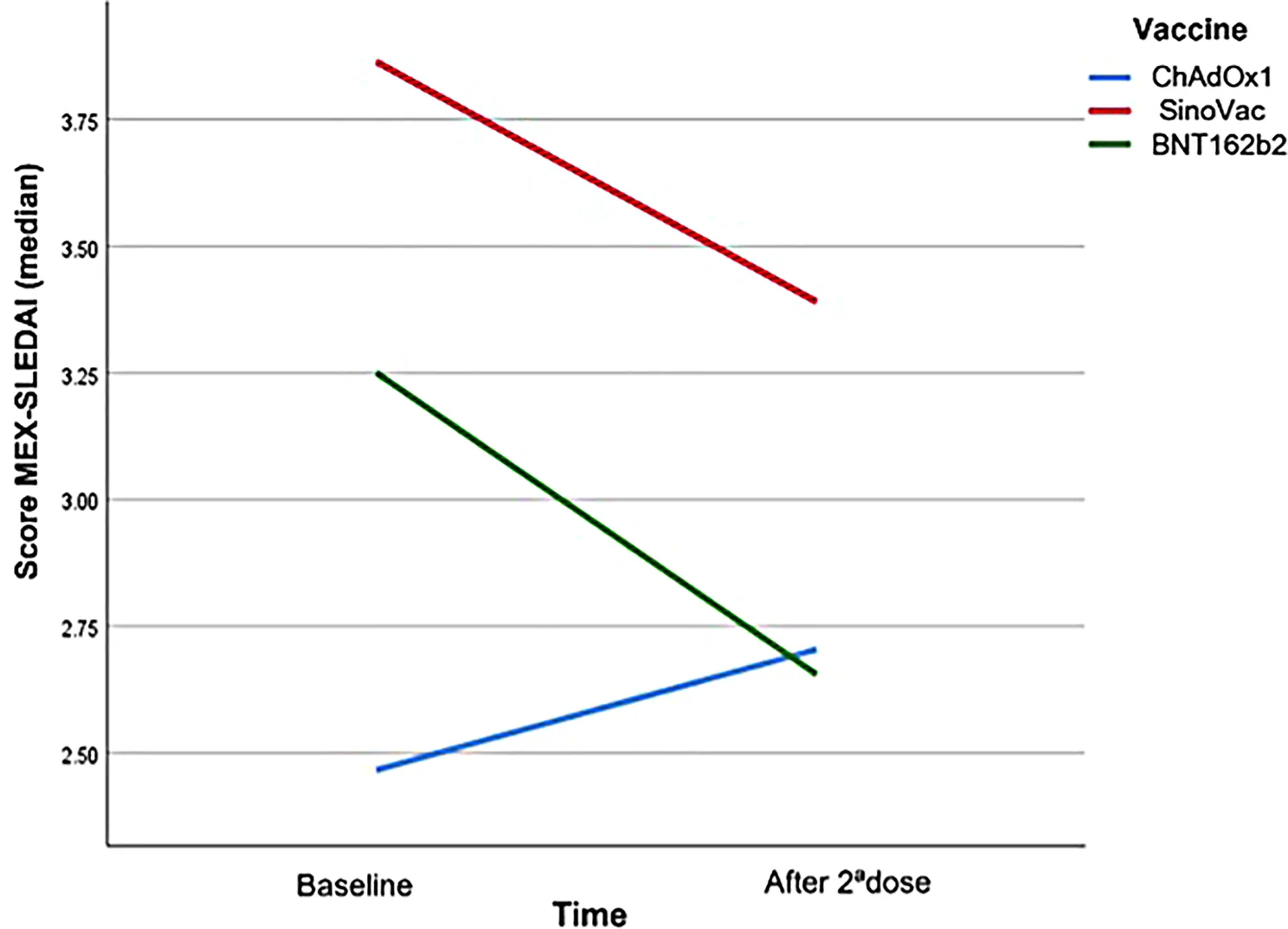
Disease activity (MEX-SLEDAI) at baseline and after second dose of different COVID-19 vaccines

Concerning disease activity, there was no difference in the mean MEX-SLEDAI score before the first dose (T1) and after the second dose of different platforms vaccines [ChAdOx1, CoronaVac and BNT162b2 (2.5 ×2.7, 3.9 × 3.4 and 3. 3 × 2.7; *p* > 0.05)]. Besides this, no difference was found between groups, considering the number of patients who needed change on the treatment between first and second evaluation (Fig. 3). The main disease activities reported were cutaneous (42%), musculoskeletal (17.8%) and renal (17.8%).

## Discussion

The current study showed that SLE patients vaccinated at least with two doses of different vaccine platforms (CoronaVac, ChadOx-1 or BNT162b2) presented a satisfactory frequency of serological response, without a significant difference between them. However, when analyzing the titer of serologic response, it was observed a higher titer in patients who had received BNT162b2 vaccine in relation to the other platforms. All vaccines were safe, without any severe event regardless of the vaccine platform. The ESAVI were mainly at site of injection; however, arthralgia, fatigue and fever were also being reported with some differences between vaccines. No significant worsening in disease activity was found after the two doses of the three different vaccine platforms.

The patients with immune-mediated rheumatic diseases are more vulnerable to the impacts of the COVID-19 pandemic. This is due not only to the condition of immunosuppression intrinsic to the disease, especially in the setting of SLE, but also to the added risk of medications and comorbidities that are more common in this context, such as diabetes mellitus, systemic hypertension, and chronic kidney disease [25]. In addition, there is a marked reduction in the humoral vaccine response against COVID-19 in patients with SLE compared to the general population [26, 27, 32, 36].

To our knowledge, there are no studies comparing the serological response of mRNA vaccine platforms (such as BNT162b2), inactivated virus (such as CoronaVac) and viral vector (such as ChadOx-1) that could validate our results. Our findings are consistent with studies conducted in the general population, where a lower humoral response was reported for inactivated or viral vector vaccines compared to mRNA vaccines [37–40].

In studies assessing the immunogenicity of different vaccine platforms in patients with systemic lupus erythematosus (SLE), findings indicate that viral vector vaccines, such as ChadOx-1, elicit a stronger immune response compared to inactivated virus vaccines like CoronaVac and COVALO. Furthermore, mRNA vaccines, including BNT162b2 and mRNA-1273, demonstrate a more robust immunogenic response than the viral vector vaccine ChadOx-1 [41]. However, the literature still lacks a direct comparison of mRNA, viral vector, and inactivated virus vaccine platforms, all of which are widely used in Brazil. The type of vaccine remains a strong independent predictor of the humoral immune response in patients with IMRDs, even when adjusted for other confounding factors, including clinical-demographic characteristics and use of immunosuppressants [37, 41, 42].

Post-vaccination seropositivity (Anti-RBD) has previously been correlated with protection against symptomatic SARS-CoV-2 infection in patients with autoimmune diseases [43]. Previous studies have also suggested a positive linear relationship between Anti-RBD (IgG) antibody levels and neutralizing antibody production [44], as well as between Anti-RBD antibody levels and clinical protection against symptomatic infections [43, 44]. Therefore, our results suggest that two doses of the BNT162b2 vaccine may confer a better serological, and potentially clinical, vaccine response in patients with SLE when compared to the CoronaVac and ChadOx-1 platforms.

The main ESAVI in our study, pain at the vaccination site, headache, arthralgia and fatigue, did not differ from those found in the healthy population [19, 22, 45]. On the other hand, we found more frequent complaints of arthralgia, which was also observed in other studies both with IMRDs and specifically in SLE [30, 31, 46–49].

Pain at the vaccination site, headache, arthralgia and fatigue were present in both the first and second doses regardless of the vaccine platform received, with the majority of ESAVI occurring after the first dose of the vaccine [50]. Studies conducted by Gerosa et al. (2022), Barbhaiya et al. (2022), Zavala-Flores (2022) and VACOLUP observed more ESAVI after the second dose [46, 47, 51, 52]. We believe that this difference is due to the fact that in these studies there was a higher frequency of BNT162b2 and ChAdOx1 vaccines, which are vaccines known to be more reactogenic, both in the first and second doses. In the studies by Gerosa et al. (2022), Zavala-Flores (2022) and Barbhaiya et al. (2022), respectively 90.0%, 100% and 59.6% received BNT162b2 and, in VACOLUP, 46% received ChAdOx1 and 41% BNT162b2, with a lower proportion of patients receiving the mRNA vaccine in the present study [46, 47, 52]. ChAdOx1 was associated with more systemic complaints, with fever being the most common symptom, a finding also observed in comparative studies in healthy populations [53, 54]. As in other studies in healthy individuals, such as a meta-analysis of 19 studies on safety and the study by Costa Clemens [55, 56], no serious ESAVI or deaths were reported. Similarly, no such events occurred in patients with SLE who received different SARS-CoV-2 vaccine platforms [46, 51, 52].

It is worth noting that previous infection with SARS-CoV-2, even if confirmed in only 23 patients, had no influence on local or systemic ESAVI and was independent of the vaccine administered, as also observed in the study by Gerosa et al. 2022 [46]. Regarding the influence of medications on ESAVI, most patients in the study were using hydroxychloroquine (79.7%) and, despite all the known benefits of the medication, its use was not found to be a protective factor against ESAVI in general or in any of the vaccines individually. Approximately 80% of the patients were considered to have a high degree of immunosuppression and were using some immunosuppressive medication. There was also no influence of these medications or the degree of immunosuppression on the presentation of ESAVI. Approximately 40% of patients were using glucocorticoids, less frequently than that observed in most studies, with a frequency of around 70% [24]. So et al. 2020, found that approximately 75% of patients were using glucocorticoids and only 55% were using some other immunosuppressant. The use of immunosuppressants by most of our patients may justify the lower frequency of glucocorticoid use. When evaluated by glucocorticoid doses greater than 7.5 mg per day, there was no difference in the presence or lesser severity of ESAVI regardless of the groups.

Regarding disease activity, the baseline MEX-SLEDAI was higher than 2.5 in all vaccine subgroups, demonstrating possible disease activity at the time of vaccination. The choice of MEX-SLEDAI was due to the absence of some tests available for performing the SLEDAI-2K. However, studies have shown a good correlation between these methods and vaccination against SARS-Cov-2 was not responsible for a significant increase in the risk of disease relapse in other studies with SLE [31, 36]. Yoshida et al., 2022 also evaluated disease activity 30 days after the second dose of vaccine, using SLEDAI-2K and did not observe a greater chance of post-vaccine relapse [57].

The main sites of post-vaccine disease activity were skin (42%), joint and kidney (17.8%). The VACOLUP study found 68% of musculoskeletal involvement and 57% of skin involvement. Although we present a higher percentage of renal involvement than that observed in the literature [36, 47], only two patients with renal activity required a change in treatment.

There are some limitations of our study: the small sample and small analysed subgroups; the exclusive evaluation of humoral immunogenicity, without analysis of cellular immunity; the exclusion of patients with a high degree of disease activity and using more potent immunosuppressants (pulse glucocorticoid therapy, rituximab or cyclophosphamide) in the immunogenicity analysis; the lack of longitudinal analysis of immunogenicity, which can decrease at different rates depending on the platform used. Our study highlights the need for studies with larger sample sizes to evaluate longitudinally the humoral response together with the cellular immunity of different vaccine platforms in patients with SLE.

## Conclusions

The study showed that patients with systemic lupus erythematosus (SLE) had similar response rates to the ChadOx-1, CoronaVac, and BNT162b2 vaccines. While the overall serological response rates were comparable among the three vaccine platforms, the BNT162b2 vaccine was noted to induce higher levels of anti-receptor binding domain (RBD) antibodies compared to the other two vaccines. Importantly, all three vaccines were found to be safe for patients with SLE, with observed ESAVI aligning with those previously documented in the literature. There were no serious events supposedly attributable to vaccination or immunization or deaths reported among the participants, suggesting that these vaccines can be safely administered to individuals with this condition.

## Electronic supplementary material

Below is the link to the electronic supplementary material.

Supplementary Material 1

## Data Availability

No datasets were generated or analysed during the current study.
