# Supplementary material for: Comparison of the immunogenicity and safety among COVID-19 vaccines ChadOx-1, CoronaVac and BNT162b2 in systemic lupus erythematosus (SLE) patients: a prospective cohort
Source: Adv Rheumatol. 2025 Nov 22;65:67. doi: 10.1186/s42358-025-00498-9 (PMC13488832; doi:10.1186/s42358-025-00498-9)
Supplement: Supplementary file 1 [file 42358_2025_498_MOESM1_ESM.docx]

**SUPPLEMENTAL MATERIALS**

**Supplemental Table 1:** Duration of events supposedly attributable to vaccination or immunization following the first dose

| **ESAVI time** | **Total** | | **ChAdOx1** | | **SinoVac** | | **BNT162b2** | | **P*** |
| --- | --- | --- | --- | --- | --- | --- | --- | --- | --- |
|  | **Mean (SD)** | **med (min;max)** | **Mean (SD)** | **med (min;max)** | **Mean (SD)** | **med (min;max)** | **Mean (SD)** | **med (min;max)** |  |
| Erythema | 3,2 (3,5) | 2 (1; 14) | 4,4 (4,2) | 3,5 (1; 14) | 1,3 (0,5) | 1 (1; 2) | 2,5 (2,1) | 2,5 (1; 4) | 0,175 |
| Bruising | 6,2 (6) | 4 (1; 17) | 5,3 (6) | 3 (1; 14) | 6 (5,7) | 6 (2; 10) | 7,7 (8,3) | 5 (1; 17) | 0,881 |
| Swelling | 3 (3,1) | 2 (1; 14) | 4,2 (3,9) | 3 (1; 14) | 2 (1,5) | 1,5 (1; 5) | 2,1 (1,8) | 2 (1; 7) | 0,272 |
| Induration | 4 (5,1) | 3 (1; 28) | 5,9 (7,5) | 4 (1; 28) | 3,1 (2) | 3 (1; 6) | 2,3 (1,9) | 2 (1; 7) | 0,395 |
| Local Pain | 1 (3,4) | 0 (0; 28) | 1,5 (5) | 0 (0; 28) | 0,3 (0,8) | 0 (0; 2) | 0,7 (0,8) | 0 (0; 3) | 0,24 |
| Skin Rash | 11,5 (10,7) | 8 (1; 28) | 6,3 (2,9) | 8 (3; 8) | 0 (0) | 0 (0; 0) | 14,6 (12,9) | 12 (1; 28) | 0,451 |
| Nausea or vomiting | 3,4 (2,4) | 3 (1; 12) | 2,6 (1,7) | 2 (1; 6) | 4,3 (2,9) | 4 (2; 12) | 3,3 (2,3) | 2 (2; 6) | 0,195 |
| Fatigue | 8,4 (9,1) | 3,5 (1; 28) | 7,2 (9,3) | 3 (1; 28) | 7,9 (8,2) | 4,5 (2; 28) | 11,3 (10,1) | 7 (2; 28) | 0,325 |
| Headache | 5,4 (5,2) | 3 (1; 28) | 4,9 (6) | 2 (1; 28) | 6,3 (3,7) | 7 (1; 14) | 5,2 (5,4) | 3 (1; 17) | 0,104 |
| Myalgia | 13 (11,2) | 9 (1; 28) | 10,7 (11,2) | 3 (1; 28) | 12 (9,7) | 10 (2; 28) | 17,3 (11,9) | 23 (1; 28) | 0,254 |
| Arthralgia | 16,2 (11,2) | 17 (1; 28) | 15,8 (12) | 18 (1; 28) | 12,7 (11) | 7 (3; 28) | 21,1 (8,9) | 27 (5; 28) | 0,256 |
| Fever | 4,2 (7) | 2 (1; 28) | 2,5 (1,9) | 2 (1; 7) | 1,5 (0,7) | 1,5 (1; 2) | 15,5 (17,7) | 15,5 (3; 28) | 0,19 |
| Vertigo | 4,6 (6) | 2 (1; 28) | 7,7 (8,3) | 5 (1; 28) | 2,9 (3,1) | 2 (1; 10) | 2,1 (1,9) | 1 (1; 6) | 0,088 |

* Teste Kruskal-Wallis: sd: standard deviation; mean: median; min: minimum; max: maximum. ESAVI: events supposedly attributable to vaccination or immunization.

**Supplemental Table 2:** General and specific events supposedly attributable to vaccination or immunization considering pre-exposed covid infection in SLE Patients n (%)

| **Dose** | **ESAVI** | | **Prior Covid Infection** | **p** | | | **Vaccine** | | | | | | | | | | |
| --- | --- | --- | --- | --- | --- | --- | --- | --- | --- | --- | --- | --- | --- | --- | --- | --- | --- |
|  |  |  |  |  |  |  | **ChAdOx1** | | | | **SinoVac** | | | | **BNT162b2** | | |
|  |  |  |  | |  | | **PC (%)** | | **p** | | **PC (%)** | | **p** | | **PC (%)** | | **p** |
| **First** | **Local** | **No** | 6 (26.1) | | 0.574 | 0 (0.0) | | 0.569^b^ | | 5 (23.8) | | 0.727^b^ | | 1 (20.0) | | > 0.99^a^ | |
|  |  | **Yes** | 17 (73.9) | |  | 4 (12.1) | |  |  | 6 (31.6) | |  |  | 7 (29.2) | |  |  |
|  | **Systemic** | **No** | 8 (34.8) | | 0.574 | 1 (16.7) | | > 0.99^a^ | | 4 (23.5) | | 0.730^b^ | | 3 (30.0) | | 0.613^b^ | |
|  |  | **Yes** | 15 (65.2) | |  | 3 (8.6) | |  |  | 7 (30.4) | |  |  | 5 (26.3) | |  |  |
| **Second** | **Local** | **No** | 9 (42.9) | | 0.139 | 1 (5.0) | | 0.595^a^ | | 6 (21.4) | | 0.234^b^ | | 2 (16.7) | | 0.662^b^ | |
|  |  | **Yes** | 12 (57.1) | |  | 2 (11.1) | |  |  | 5 (45.5) | |  |  | 5 (31.3) | |  |  |
|  | **Systemic** | **No** | 11 (52.4) | | 0.922 | 1 (5.9) | | > 0.99^a^ | | 6 (25.0) | | 0.718^b^ | | 4 (30.8) | | 0.670^b^ | |
|  |  | **Yes** | 10 (47.6) | |  | 2 (9.5) | |  |  | 5 (33.3) | |  |  | 3 (20.0) | |  |  |

^a^Chi-square test. ^b^Fisher’s Exact Test. PC: previous covid infection. ESAVI: events supposedly attributable to vaccination or immunization.

**Supplemental Table 3:** Events supposedly attributable to vaccination or immunization according immunosuppressants use in SLE patients n (%)

| **Dose** | **ESAVI** | | **Level of immunosuppression** | | | |  |
| --- | --- | --- | --- | --- | --- | --- | --- |
|  |  |  | **No** | **Low** | **High** | **p**^a^ |  |
| **First** | **Local** | **No** | 7 (35.0) | 0 (0.0) | 28 (30.8) | 0.857 |  |
|  |  | **Yes** | 13 (65.0) | 1 (100) | 63 (69.2) |  |  |
|  | **Systemic** | **No** | 3 (15.0) | 0 (0.0) | 31 (34.1) | 0.744 |  |
|  |  | **Yes** | 17 (85.0) | 1 (100) | 60 (65.9) |  |  |
| **Second** | **Local** | **No** | 8 (42.1) | 1 (100) | 52 (59.8) | 0.166 |  |
|  |  | **Yes** | 11 (57.9) | 0 (0.0) | 35 (40.2) |  |  |
|  | **Systemic** | **No** | 9 (47.4) | 1 (100) | 46 (52.9) | 0.896 |  |
|  |  | **Yes** | 10 (52.6) | 0 (0.0) | 41 (47.1) |  |  |

^a^Fisher’s Exact Test. ESAVI: events supposedly attributable to vaccination or immunization.

**Supplemental Table 4:** Events supposedly attributable to vaccination or immunization according prednisone dose and type of vaccine use in SLE Patients n (%)

| **Dose** | **ESAVI** | | **Total** | | **Vaccine** | | | | | | |
| --- | --- | --- | --- | --- | --- | --- | --- | --- | --- | --- | --- |
|  |  |  |  |  | **ChAdOx1** | | | **SinoVac** | | **BNT162b2** | |
|  |  |  | **Prednisone** ≥ **7,5mg** | **p** | | **Prednisone** ≥ **7,5mg** | **p** | **Prednisone** ≥ **7,5mg** | **p** | **Prednisone** ≥ **7,5mg** | **p** |
| **First** | **Local** | **No** | 9 (64.3) | 0.693^a^ | | 1 (25.0) | 0.603^a^ | 6 (75.0) | > 0.99^b^ | 2 (100) | > 0.99^b^ |
|  |  | **Yes** | 18 (58.1) |  |  | 6 (46.2) |  | 5 (71.4) |  | 7 (63.6) |  |
|  | **Systemic** | **No** | 11 (68.8) | 0.373^a^ | | 1 (25.0) | 0.603^a^ | 6 (75.0) | > 0.99^b^ | 4 (100) | 0.228^b^ |
|  |  | **Yes** | 16 (55.2) |  |  | 6 (46.2) |  | 5 (71.4) |  | 5 (55.6) |  |
| **Second** | **Local** | **No** | 17 (60.7) | 0.772^a^ | | 2 (25.0) | 0.608^a^ | 10 (71.4) | > 0.99^b^ | 5 (83.3) | 0.559^b^ |
|  |  | **Yes** | 9 (56.3) |  |  | 3 (42.9) |  | 2 (100) |  | 4 (57.1) |  |
|  | **Systemic** | **No** | 15 (65.2) | 0.387^a^ | | 3 (50.0) | 0.580^a^ | 7 (63.6) | 0.245^b^ | 5 (83.3) | 0.559^b^ |
|  |  | **Yes** | 11 (52.4) |  |  | 2 (22.2) |  | 5 (100) |  | 4 (57.1) |  |

^a^Chi-square test; ^b^Fisher’s Exact Test. ESAVI: events supposedly attributable to vaccination or immunization.
